# The Impact of Reality Therapy on Metacognition, Stress and Hope in Addicts

**DOI:** 10.5539/gjhs.v6n6p281

**Published:** 2014-09-23

**Authors:** Mehdi Karimyar Jahromi, Leili Mosallanejad

**Affiliations:** 1 Research Center for Social Determinants of Health, Jahrom University of Medical Science, Jahrom, Iran; 2 Mental Health Nursing Department, Research Center for Social Determinants of Health, Jahrom University of Medical Science, Jahrom, Iran

**Keywords:** reality therapy, metacognition, stress, hope, drug addiction

## Abstract

**Introduction:**

Drug dependence is a major problem in our country and in international level. Reality therapy is an internal control system that determines why and how to choose the options that are set for our lives. This study aimed to investigate the effect of reality therapy on metacognition, stress and decline in hope in drug addicts.

**Material and methods:**

This study is quasi-experimental study. Samples were chosen convenient among 60 drug addicts in Jahrom in 2012. Samples were randomly divided into two groups (each group = 30) of intervention and control groups. Control group received usual care and experimental group received reality therapy.

**Results:**

Results revealed that there was significant difference between the mean scores of metacognition and hope before and after the intervention of experimental group (p < 0.05). Also, there was statistically significant difference between the two groups from these two variables (p < 0.05). But, Stress level wasn’t statistically different in two groups.

**Discussion:**

Reality therapy is a method that emphasizes the accountability and the current behavior of individuals. Setting negative emotions, reality therapy promotes metacognition and, through increasing accountability to self-behavior improves hope.

## 1. Introduction

Drug dependency is a major problem in our country and in international level, since on one hand, it threatens the health of society and on the other hand, it is associated with many crimes and diseases such as AIDS (Madani & Emadi, 2003). Drug misuse and addiction is a serious illness which ruin the economy improvement, communications and the individual’s occupation (Carlo et al., 2004). Thus, there are multiple medical treatments and various psychological treatment methods in this field (Carlo et al., 2004; Katibaee et al., 2010; Ahmadi & motamed, 2002). Reality therapy is an internal control system which clarifies how and why to choose options which determine the direction of our lives (Wubbolding, 2006; Aghaee, 2002). This theory is widely used for the treatment of addictive disorders (such as drugs, sex, food, work) (Kim et al., 2008). Reality therapy method, which later altered to selection theory, emphasizes encountering accepting responsibility and moral judgment about right and wrong of ethics (Mottern & Mottern, 2006). Reality therapists believe that the drug addicts choose their behavior as a way to solve the failure caused by unpleasant relations (Carey, 2007).

Reality therapy is a method based on common sense and emotional engagement. This theory is based on identity quality, its formation and change since human has unaccountable behavior when encountering anxiety and discomfort (Glaser, 2009). Gaining control over the choices and accepting the responsibility in this field should be the subject of the treatment. Modifying different psychological aspects of individual, reality therapy helps them to deeply face the realities of their behavior and choices and recognizes that they are responsible for their miseries and misfortunes (Carlo et al., 2004).

Therefore, reality therapy provides the context for mental and psychological health promotion by direct impact on various mental, psychological, and personality aspects (Kim, 2008). Metacognition is a personal knowledge about self cognitive processes which consist of processing, organizing, and coordinating of all of them. Metacognition is a multidimensional concept. This concept consists of knowledge, processes, and strategies which evaluate, supervise, or control cognition. Metacognition skill is a modern scholar area which is closely related to anxiety (Wells, 2002). Awareness of human cognitive processes and finding ways to enhance and improve these abilities, metacognition has gained the attention of educational experts (King, 1994). Schraw and moshman (1995) believe that three cultural, public interaction and self-constructing factors involve in creating and founding individual’s metacognitive theories (Schraw & Moshman, 1995). Metacognition is one of the variables which is disrupted through drug misuse and may be particularly related to the analysis of drugs dependent cognition. From the metacognitive perspective, drug abuse creates significant rapid changes in cognitive events such as feelings, thoughts or memories.

Drugs and psychotropic drugs may affect cognitive events directly for instance, relaxation, avoidance, running away from painful cognitions, and awareness and attention or indirectly for example, feelings of attachment, suppressed astonishment and assessment by changes in beliefs and attitudes toward avoiding cognitive events. These cognitive changes may result in powerful negative and positive reinforcements of drugs use (Casey & Jones, 2010). In addition, the use of drugs not only creates beliefs and positive expectations about the effects of drugs and psychotropic drugs, but also helps to gain knowledge and awareness about the cognitive consequences. Lack of using drugs when the person is willing to use it, may be related to specific beliefs and attitudes toward the effectiveness of related cognitive events (Toneatto, 1999). Stress is one of the problems and disorders which is revealed by the human improvement in various life aspects. When the person cannot deal with the situation and this situation somehow makes discomfort, it leads to stress. An important feature of addiction which is the psychological dependence on the effects of addictive drugs, takes away favorable conditions for the development of the individual abilities. Apathy, inefficiency, lack of accountability and the negative attitude of society to the addicted person all are the features that endanger the addicted person from the mental perspective. Thus, the addicted people are often involved in a variety of emotional symptom including stress (Sohrabi, 2007). Since the addicted person has no efficiency, the feeling of helpfulness and efficiency is formed less. Therefore, stress attacks him more and this negatively affects the capability, efficiency of the addicted person and declines his creativity and self-esteem (Sohrabi, 2007). Hope is one of the basic principles of balance and mental strength gains which determines the life achievements. Hope is the ability to believe a better feeling of future. Hope with its penetrating power stimulates activity system to help the system to gain new experiences and creates new forces to the organism (Gullo & Dawe, 2008). Thus, hope makes human to attempt and approaches them to higher levels of psychological and behavioral performances and is a sign of mental health (Islami, 1998). Considering the destructive effects of drug on metacognition, making stress and declining hope and the direct effect of these cases on prognosis of addiction treatment and lack of enough study in this field, the current study was carried out to investigate the effect of reality therapy on these three factors in people referring to one of the NA centers.

## 2. Material and Methods

This study was before and after quasi-experimental study with control group. Sampling was carried out simply by picking up 60 addicts who referred to Shafa NA Center in Jahrom in the fall and the winter of 2012. Samples had no history of psychological-mental disorders, alcohol and psychotropic drug use and it was their first attempts to quit. All samples were male and aged between 20 to 35 years.

Samples were randomly assigned into two groups of intervention and control (each group 30).

Checklist associated with metacognition evaluation, stress and hope were completed through interview by the first psychologist in both experimental and control groups. To evaluate metacognition, a 20-question standard questionnaire of Anil and Abedi (1996) was applied which investigates metacognition in 4 elements of awareness, cognitive strategy, planning, and self reviewing. Measuring scale was educational however; the range varies from 1 to 4. This questionnaire has been applied in several studies and the reliability has been reported 0.70 to 0.83 using Cronbach’s alpha. To evaluate stress index, the standard questionnaire of Cohen et al. (1983) was used. This scale has 14 items and responses are based on Likert scale. Consistency of reliability coefficient of internal scale was achieved through two-range Cronbach’s alpha coefficients from 0.84 to. 0.86 in the two groups of students and a group of smokers in the quitting plan.

The Scale of Hope Adult Schneider (SAHS) also measured through (Schneider et al., 1991) questionnaire that was designed by (Snyder & Lopez, 2007) to measure hope, contains 14 expressions and is run as a self-assessment. From among these, four expressions measure agent thought, four expressions measure strategic thought, and four expressions are distractors. So, the questionnaire measures two subscales: agent and strategy. The coefficient of validity and reliability of the test was reported 91%. Internal consistency reported 0.74-0.84 also Test-retest reliability was 0.80. This number during the periods of more than 8-10 weeks was higher than this value (Snyder et al., 2007).

In Iran this scale has been standardized and has achieved a good reliability. For example, in the study of Alavi (2006) *Cronbach’s Alpha* Reliability Coefficient was obtained 0.75 and it was achieved 0.62 in the study of Nasiri and Jokar (2008).

Then control group received usual care of psychological center and experimental group received reality therapy during nine sessions by the psychologists. The first session include familiarity with the concept of reality therapy and emotional engagement with members of the group, the second session was recognition of their identity and various kinds of identities and characteristics of achievement and failure identities, the third session was investigating how to accept their responsible behavior and getting to know other members and the importance and necessity of accountability in life, the fourth session was familiarity with anxiety from the viewpoint of reality therapy and relaxation training for anxiety control, the 5th session was expressing the vital and effective requirements in life and their capabilities to choose the best method to achieve basic needs, the 6th session was familiarity with how to plan problem solving and plan for their present lives, the seventh session was understanding the commitment to performing completed plans, the eighth session was familiarity with how to decline excuses related to plans and selected programs, and the ninth session was familiarity of members with the effect of punishment in absence of creating a good relationship. The sessions meet once a week for 45-60 minutes and after completion of the course, the checklists related to metacognition, stress, and were completed through interview by the first psychologist in both groups.

This study confirmed by Ethical Committee in Jahrom University of Medical sciences.

## 3. Results

This study conducted with 60 drug addicts referring to NA centers in two control and experimental groups (each group 30). All the samples were male; the mean age of experimental group was 21.7±3.2 and the control group was 21.95+1.8. The participants of both groups were 17 (56.6%) and 20 (66.6%) married and 21 (70%) and 19 (56.6%) had academic education (Associated and Master’s degrees) respectively. In both groups, 12 (40%) were unemployed. Duration of addiction in both control and experimental groups were 3.09±0.4 and 2.8±0.78 year respectively. All used opium and its derivatives. Most participants in both groups (56.6%) of control group and 70% of experimental group) had poor economical condition. No one of them have any somatic and moderate to severe somatic or psychological disorder in clinical examination by physician. There was no significant difference from above mension between two groups (Table 1).

**Table 1:** Demographic distribution in two groups

Distribution	Group	Demographic
**Age**	Experiment	21.7±3.2
	Control	21.95±1.8

**Married**	Experiment	19 (63.3%)
	Control	20 (66.6%)

**Literacy**	Experiment	21 (70%)
	Control	19 (63.3%)

**Unemployment**	Experiment	15 (50%)
	Control	14 (40%)

**Poor economic status**	Experiment	22 (73.3%)
	Control	23(76.6%)

All p value is not sig (NS).

Normality of variable tested in groups. Results showed equality of characteristic distribution by the ***Kolmogorov-****Smirnov* ***test*** *(KS-**test**)*. Considering metacognition, the mean scores of pre reality therapy in both experimental and control groups were 36.01±4.3 and 37.4±5.12 and post intervention were 40.02±0.83 and 36.9±4.1 respectively. Paired t.test reveals a significant relationship between pre and post intervention of the experimental group (P < 0.05); however, the relationship between pre and post intervention of the control group wasn’t significant (P > 0.05). The results indicate that metacognition situation after the intervention experiment group comparing with control group was more appropriate.

Considering stress, the mean scores in control group before and after the intervention were 47.33±1.2 and 46.9±0.54 and in experimental group were 48.05±0.37 and 49.1±0.66 respectively. Paired t-test showed that there was no significant difference between the two groups before and after the intervention (P > 0.05).

Considering life expectancy, the mean scores of experimental group before and after the reality therapy were 22.4±1.38 and 33.7±0.74 and in control group were 20.4±0.6 and 21.09±0.3 respectively. The paired t.test revealed that there was a significant difference between the scores of before and after in experimental group (P < 0.05); however, the difference was not significant in control group. According to Student t.test, the difference between mean score before the intervention wasn’t significant in both groups (P > 0.05) (Table 2).

**Table 2:** The differences between indicators in experiment and control group

Aspects	Groups	Before mean (SD)	After mean(SD)	Statistical tests	

				P from paired t-test	P value from student t-test
Metacognition	Intervention	4.3±36.01	0.83± 40.02	0.01	0.001
	Control	5.12± 37.4	4.1±36.9	0.54	

Stress	Intervention	0.37±48.05	0.66±49.1	0.74	0.104
	Control	1.2±47.33	0.54±46.9	0.44	

Life expectancy	Intervention	1.38±22.4	0.74±33.7	0.001	0.03
	Control	0.6±20.4	0.3±21.09	0.34	

Pearson test showed that there was a significant relationship between the scores of metacognition and education level (r=0.66), stress and education level (r=0.91). Also there was a correlation between stress and employment (r=0.76), metacognition and employment (r=0.81) and metacognition and economic level (r=0.90) (Table 3).

**Table 3:** Relationship between variables

	Literacy	employment	economic level
Stress	(r = 0.91)	(r = 0.76)	
Metacognition	(r = 0.66)	(r = 0.81)	(r = 0.90)

All p value is significant (P < 0.05).

r: correlation.

## 4. Discussion

The results of this study showed that reality therapy had been effective in metacognition situation and but had no effect on declining stress level in the drug addicts.

No study was conducted to investigate the effect of reality therapy on metacognition but there are similar results in other groups. In a study conducted by Panah et al. (2004), group reality therapy had positive impact (inhibitive) on the students attitudes toward smoking of first year high school students.

The results of the study of Hosseinpour et al. (2009) revealed that educational effectiveness of accountability in Glosser reality therapy approach would lead to reduction of students” identity crisis. The study of Saed et al. (2010) indicated that proper interventions and treatment plans provided the possibility to reduce drug dependence by correcting metacognition factors.

According to metacognition approach dependence to drugs in short term functions as an adaptive coping strategy to regulate negative excitements. But in the long term it is considered maladaptive, since it can cause dependence and produce negative excitement (Spade, 2008). Due to ineffective metacognitive beliefs, people with drug abuse disorder (for example beliefs about the need to control thoughts or negative beliefs about uncontrollability and danger of thoughts), are affected by emotional distress.

Excessive control over the mind and thought in the drug addicted would lead to increase access to negative information and consequently fails to control thoughts. The created negative excitements cause that the person resorts to ineffective strategies such as drug abuse for long term to decrease disturbing thoughts and negative excitements and be vulnerable toward dependence disorder (Saed et al., 2010). Reality therapy is a combination of techniques, approaches, and tools which help people to move from ineffective to effective behavior, destructive instead of constructive selections, and most importantly, satisfied to unsatisfied lifestyle and like other psychological approaches, it creates change by its own special approach in the behavior of clients (Glaser, 2009). Metacognitive beliefs play a role in vulnerability toward dependence disorder and correcting them would decrease vulnerability in drug dependence (Spade, 2008).

The results of the present study indicate no significant effect of reality therapy on stress. The results of the study of Jafari et al. (2009) demonstrated that educating coping skills in prevention of addiction relapse was effective in increasing resiliency and reducing the effects of stress on drug-dependent individuals.

Prinzela (2006) examined the intervention effect of reality therapy based on choice theory in PTSD patients. The results of this study indicated that reality therapy interventions were effective in reducing thought rumination in these patients. In the study of Lotfi and Askari (2009) behavioral-cognitive treatment has been effective in changing psychological status of crack addicts such as reducing anxiety. In the study of Hosseinian et al. (2008) supportive mental health has been effective in the quality of life and health of male addicts and improving different health factors (physical, psychological, independence, environmental and personal beliefs), and reducing stress. In another study the results of the study of Saed et al. (2009) revealed that psychotherapy approach based on cognitive and metacognitive treatments could reduce stress and drug dependence in addicts. The different results of the present study are probably because stress and the involving factors such as physical and psychological dependence of addicts are different from others (Curry et al., 2006), and the second is that mere reality therapy cannot be substituted by education of communication skills or supportive psychotherapy, particularly in drug addicts. Thus, more studies must be conducted in this field.

Considering the effect of reality therapy on life expectancy, no studies have been carried out, but the conducted studies in other groups indicated the effect of it on reducing stress which is in line with the present study. The study of Pasha and Amini (2008) demonstrated that reality therapy increased and reduced anxiety among wives of martyrs. The studies of Klug (2006), Barens and Parish (2006), and Davidson et al. (2007) showed that reality therapy has increased life expectancy.

In our study, there is not any significant difference in stress level between two groups, also in experiment group slightly raised. This change may be from short term effects of intervention, but it should be evaluate long term effects of intervention in these patients.

Limitations of this study were non-random selection of the samples due to the limited number of administrative problems. Another restriction was lack of consideration of other factors involved in measured variables such as sociocultural conditions and other effective factors that caused addiction. It is suggested that the role and contribution of reality therapy are investigated alone or in combination with other supportive and psychotherapy interventions in metacognitive beliefs, negative excitements, and thought suppression in drug abuse disorder much broadly and more confidently.

## 5. Conclusions

Reality therapy is a method which focuses on accountability and present behavior of individuals. Hope is optional and depends on human behavior. so, it provides opportunities that individual learn proper behavior to meet their needs, also this approach may be leading to the increase of life expectancy in addicted patients. We suggest group psychological approach as hope therapy in side medical treatments.
